# The Stability, Microstructure, and Microrheological Properties of *Monascus* Pigment Double Emulsions Stabilized by Polyglycerol Polyricinoleate and Soybean Protein Isolate

**DOI:** 10.3389/fnut.2020.543421

**Published:** 2020-12-15

**Authors:** Duoxia Xu, Boyan Zheng, Yixin Che, Guorong Liu, Yingmao Yuan, Shaojia Wang, Yanping Cao

**Affiliations:** Beijing Advanced Innovation Center for Food Nutrition and Human Health (BTBU), School of Food and Health, Engineering and Technology Research Center of Food Additives, Beijing Higher Institution Engineering Research Center of Food Additives and Ingredients, Beijing Technology & Business University, Beijing, China

**Keywords:** *Monascus* pigment, double emulsions, microrheological properties, stability, microstructure

## Abstract

*Monascus* pigment is a natural food pigment and is commonly used for coloring and as antiseptic of cured meat products, confectionery, cakes, and beverages. However, *Monascus* pigment is sensitive to environmental conditions. The main aim of this study was to investigate the effect of polyglycerol polyricinoleate (PGPR) and soy protein isolate (SPI) on the particle size, zeta potential, physical stability, microstructure, and microrheological properties of *Monascus* pigment double emulsions. The effects of ionic strength, heating, and freeze thawing treatment on the stabilities of *Monascus* pigment double emulsions were also characterized. It was found that the optimum PGPR and SPI concentrations for fabricating *Monascus* pigment double emulsion were 3.6 and 3.0 wt%, respectively. The fabricated *Monascus* pigment double emulsion was composed of fine particles with narrow and uniform size distributions. Microrheological property results suggested that the elastic characteristic of the *Monascus* pigment double emulsion was dominated with increasing PGPR and SPI contents. It was mainly due to the increased collision and interaction between the droplets during the movement resulting in force increasing. *Monascus* pigment double emulsions with <5 mM CaCl_2_ prevented calcium to destroy the physical stability of emulsions, while *Monascus* pigment double emulsions with more than 10 mM CaCl_2_ formed creaming. After freeze thawing treatment, creaming occurred in *Monascus* pigment double emulsion. However, it was stable against heating treatment due to heating leading to a dense network structure. It could be contributed to the practical applications of *Monascus* pigment double emulsions in food products.

## Introduction

In recent years, people are more aware of the importance of health; these findings make natural pigments become more popular as food colorants (1). *Monascus* pigment is a natural food pigment and is commonly used for coloring and as antiseptic of cured meat products, confectionery, cakes, and beverages (2). *Monascus* has been proven to have good biological function in various fields, including lowering blood lipids, lowering blood pressure and cholesterol levels, anti-inflammatory and anti-cancer activity, anti-mutation, immunity, anti-depression, prevention of arteriosclerosis, and other biological activities (3, 4).

However, *Monascus* pigment is sensitive to environmental conditions such as heating, basic or acidic pH, light, and oxygen (5). Therefore, its application in food is limited. There are some studies about the stability of *Monascus* pigment. Vendruscolo et al. (6) reported the thermal stability of natural pigments produced by *Monascus* ruberin-submerged fermentation. Jian et al. (7) used gum arabic as a stabilizer for improving *Monascus* pigment water solubility under acidic conditions through the formation of *Monascus* pigment–gum arabic complexes. It was found that the study of improving the stability of *Monascus* pigments was limited (8). Little attention has been paid to improve the stability of *Monascus* pigments by food delivery system.

Water-in-oil-in-water (W/O/W) emulsions are complex liquid dispersion systems known also as double emulsions, in which a water-in-oil emulsion dispersed in a second continuous water phase (9–11). It is reported that double emulsions can be used to protect and control release of bioactive compounds (12, 13). Therefore, double emulsions have good prospect in the application of food, pharmacy, and cosmetic fields (14, 15). In previous studies, natural pigments such as β-carotene and curcumin were encapsulated in emulsions for stabilization studies (16, 17). In order to improve the properties of food colorants, beetroot betalains have also been encapsulated in W/O/W emulsions, leading to stable pink-colored double emulsions (14). Using the inner water phase of W/O/W emulsions for encapsulation of *Monascus* pigment could isolate them from the detrimental surrounding aqueous environment; in order to achieve this, a stable formulation needs to be developed.

Therefore, more comprehensive studies are required to fabricate *Monascus* pigment double emulsions. Hydrophobic emulsifier polyglycerol polyricinoleate (PGPR) and hydrophilic emulsifier soy protein isolate (SPI) have been shown to be effective for the fabrication of W/O/W emulsions for encapsulation of pigment (18). The main aim of this study was to investigate the effect of different concentrations of polyglycerol polyricinoleate (PGPR) and soy protein isolate (SPI) on the physical stability, microstructure, and microrheological properties of *Monascus* pigment double emulsions. The effects of ionic strength, heating, and freeze thawing treatment on the stability of *Monascus* pigment double emulsions were also characterized. The present study could demonstrate the potential of W/O/W emulsions as an effective delivery system for the stabilization of *Monascus* pigment. Ultimately, this work could be contributed to the practical applications of *Monascus* pigment double emulsions in food products.

## Materials and Methods

### Materials

*Monascus* pigment was obtained from Guangzhou Tianyi Biologic Technology Co., Ltd (Zhanjiang, China). The hydrophilic emulsifier soy protein isolate (SPI, protein ≥ 90.0 wt%) was purchased from Shanghai Yuanye Biologic Technology Co., Ltd (Shanghai, China). The hydrophobic emulsifier polyglycerol polyricinoleate (PGPR) was purchased from Meida Food Co., Ltd (Beijing, China). Soybean oil (China Kerry Grain and Oil Co., Ltd.) was purchased from a local supermarket and used without further purification. All other chemicals were of analytical grade.

### Preparation of *Monascus* Pigment Double Emulsions

#### Preparation of Primary Water-in-Oil (W/O) Emulsion

**Figure 1:**
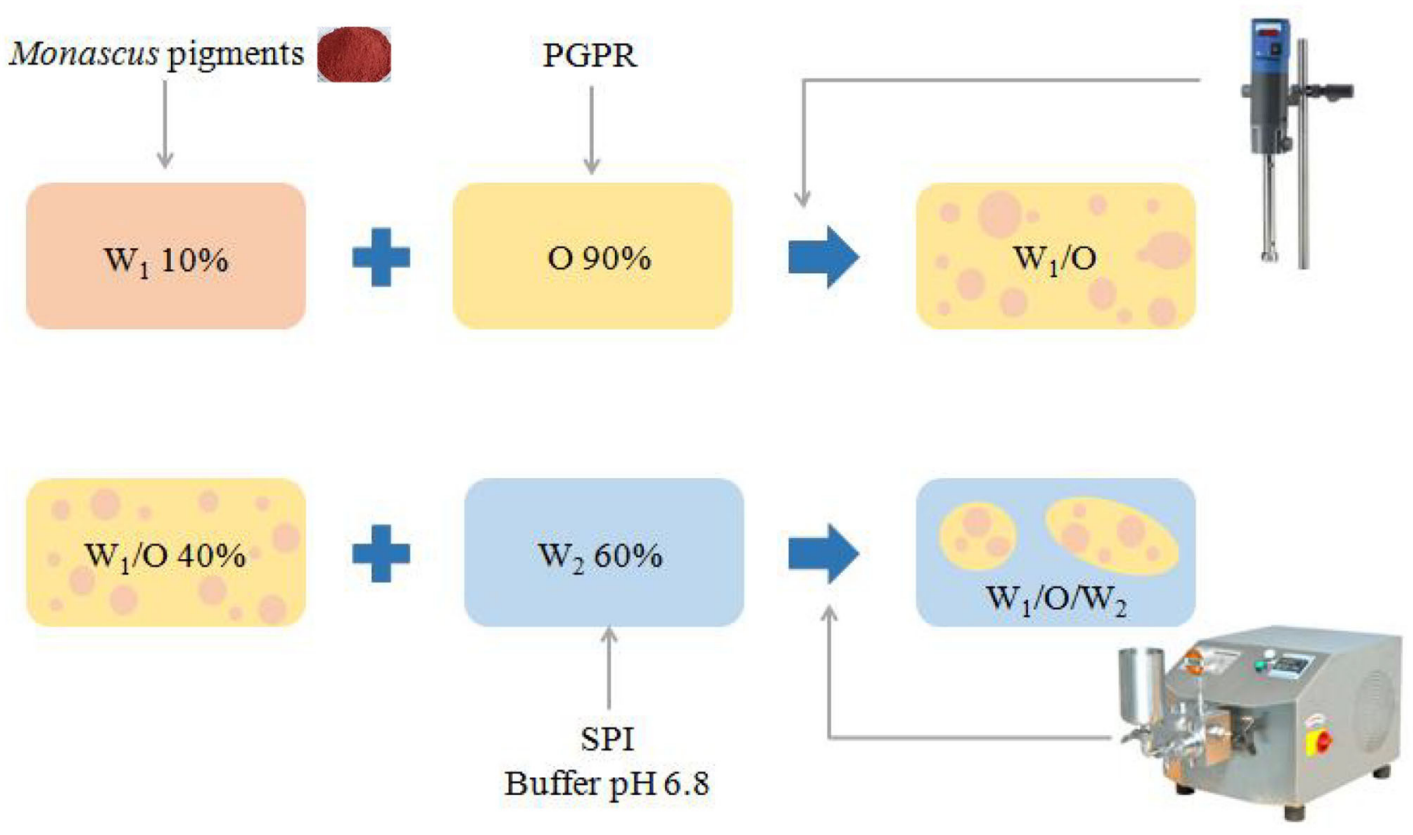
The process of preparing *Monascus* pigment double emulsions.

*Monascus* pigment double emulsions were made in a two-step procedure as shown in Figure 1. The primary W/O emulsion was formed with 10 wt% inner water phase and 90 wt% oil phase. For the inner water phase, 0.5 wt% *Monascus* pigment was dissolved in ultrapure water at 35°C for 1 h. The oil phase was prepared by dispersing different concentrations of PGPR in soybean oil and mixed it with a magnetic stirrer at 50°C for 2 h. W/O emulsions were prepared with an Ultra-Turrax homogenizer (IKA T25, Germany) at 15,000 rpm for 10 min at room temperature.

#### Preparation of *Monascus* Pigment Double Emulsion

For the outer water phase, different concentrations of SPI were dispersed in phosphate buffer at pH 6.8 and mixed it with a magnetic stirrer at 50°C for 2 h. The *Monascus* pigment double emulsion consists of 40 wt% W/O inner emulsion and 60 wt% aqueous phase. The primary *Monascus* pigment W/O emulsion and the external aqueous phase containing different concentrations of SPI were sheared at 11,000 rpm for 10 min, at room temperature. The coarse emulsions were subsequently homogenized using a homogenizer (GYB30-6S, Donghua factory, Shanghai, China) at an operational pressure of 50 MPa three times, respectively.

In order to investigate the effect of different contents of emulsifiers (PGPR & SPI) on the fabrication of *Monascus* pigment W/O/W emulsion, the final double emulsions with different contents of emulsifiers PGPR ranging from 0.9 to 5.4 wt% and SPI ranging from 0.15 to 3.0 wt% were designed.

#### Addition of Different Contents of CaCl_2_ in the *Monascus* Pigment Double Emulsions

The influence of ionic strength on the *Monascus* pigment double-emulsion physical stability was studied. Different concentrations of CaCl_2_ solution (0, 10, 20, 40, 100 mM) were prepared and then mixed with the *Monascus* pigment double emulsions (3.6 wt% PGPR and 3.0 wt% SPI) in a 1:1 ratio (v/v).

#### Heating and Freeze Thawing Treatments on the *Monascus* Pigment Double Emulsions

To investigate the influence of temperature on the stability of *Monascus* pigment double emulsions, the *Monascus* pigment double emulsions (3.6 wt% PGPR and 3.0 wt% SPI) diluted with phosphate buffer in a 1:1 ratio (v/v) were heated at 90°C for 30 min by thermostat water bath. For the freeze thawing treatment, the *Monascus* pigment double emulsions were frozen at −18°C for 22 h then thawed at 40°C for 2 h by thermostat water bath.

#### Particle Size Measurement

The average droplet size of *Monascus* pigment double emulsions was determined according to the method of Wang et al. (19) by dynamic light scattering (DLS) using a Zetasizer Nano-ZS90 (Malvern Instruments, Worcestershire, UK) at a fixed detector angle of 90°. *Monascus* pigment emulsions were diluted using 1.0 mM phosphate buffer solution at pH 6.8 to minimize multiple scattering effects prior to each measurement. The measured time correlation functions were analyzed by Automatic Program equipped with the correlator. The results were described as mean particle diameter (size, nm) and particle size distribution.

#### Zeta Potential

The zeta potential of the *Monascus* pigment double emulsions was measured by using Zetasizer Nano-ZS90 (Malvern Instruments, Worcestershire, UK). The zeta potential was determined by measuring the direction and velocity of droplet movement in the applied electric field. All *Monascus* pigment double emulsions were diluted 50 times using 1.0 mM phosphate buffer solution at pH 6.8 to avoid multiple scattering effects. After loading the samples into the instrument, they were equilibrated for about 120 s before particle charge data was collected over 11 continuous readings (20).

#### Physical Stability Analyzed by LUMisizer

The physical stability of the *Monascus* pigment double emulsions with different ratios of SPI and PGPR were measured with the LUMisizer (L.U.M. GmbH, Berlin, Germany), an instrument employing centrifugal sedimentation to accelerate the occurrence of instability phenomena such as flocculation, sedimentation or creaming (21, 22). The integration graph shows the percentage of light absorbance per hour described as the “instability index.” The physical stability of the *Monascus* pigment double emulsions can be reflected by the instability index, which is the integrated transmission profile against the measuring time; the higher the instability index, the lower the stability. The instrumental parameters used for the measurement were as follows: volume, 0.4 mL of dispersion; 2,500 rpm; time, 7,620 s; time interval, 30 s; temperature, 25°C (23).

#### Microstructure

In this experiment, the microscopic particles of the *Monascus* pigment double emulsion were directly observed by confocal laser scanning microscopy (FV1200, Olympus, Japan). The SPI in the *Monascus* pigment double emulsions was dyed with Nile blue at a ratio of 0.025% (w/w), and the soybean oil was dyed with Nile red at a ratio of 0.005% (w/w). The fluorescence signal from the Nile red and Nile blue dyes was obtained, respectively, by exciting the samples using laser sources of 488 and 637 nm, and collecting wavelengths 590–650 nm (24). All microstructures were observed with a 60 × objective lens (oil immersion).

#### Microrheological Property

The commercial Rheolaser Master (Formulaction, l'Union, France) used for the measurements of the microrheological property of *Monascus* pigment double emulsions is based on diffusing wave spectroscopy (DWS). The mean square displacement (MSD) curves were calculated from the dynamic speckle images, scattering by the sample as a function of time (25). The *Monascus* pigment double emulsions was placed into flat-bottomed cylindrical glass tubes (140 mm height, 16 mm diameter). The obtained data were calculated by the software Rheosoft Master 1.4.0.0 and expressed as solid-liquid balance (SLB) value.

#### Statistical Analysis

All *Monascus* pigment emulsions were prepared in triplicate, and all measurements were performed three times. Data were subjected to analysis of variance (ANOVA) using the software package Origin 8.5 for Windows.

## Results and Discussion

### Effect of PGPR on the *Monascus* Pigment Double Emulsions

#### Particle Size

**Figure 2:**
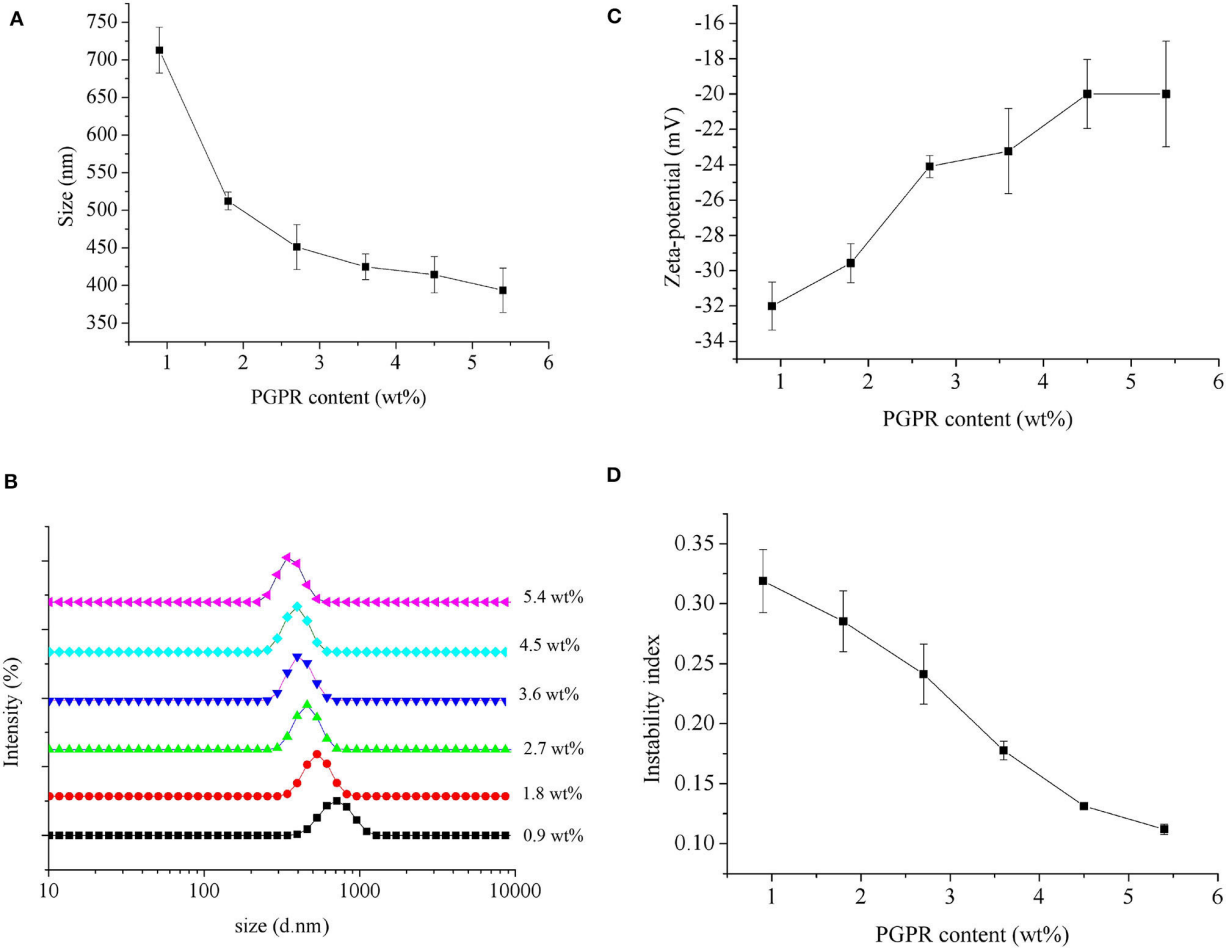
Influence of PGPR concentrations on the mean particle size **(A)**, particle size distribution **(B)**, zeta potential **(C)**, and instability index **(D)** of *Monascus* pigment double emulsions.

The effect of different concentrations of PGPR on the average droplet size of *Monascus* pigment double emulsion is shown in Figure 2A. There was a significant decrease in the average droplet size from 713.0 ± 30.7 to 451.0 ± 30.0 nm with increasing amount of PGPR from 0.9 to 2.7 wt%, after which the average droplet size decreased from 424.8 ± 17.1 to 393.5 ± 29.6 nm with the increase of PGPR concentration from 3.6 to 5.4 wt%. The effect can also be seen in the droplet size distributions (Figure 2B). The *Monascus* pigment double emulsion contained a single peak at each PGPR concentration. The peak shifted to the left and decreased in intensity with increasing PGPR concentrations. For the lower PGPR concentration tested (0.9 and 1.8 wt%), the *Monascus* pigment emulsions showed a narrow size distribution, peaks corresponding to bigger sizes. Compared to *Monascus* pigment double emulsions with 2.7 wt% PGPR, the sample with 3.6 wt% PGPR was composed of fine particles with a narrow and uniform size distribution. At higher PGPR concentrations (4.5 and 5.4 wt%), the peaks of *Monascus* pigment emulsions shifted slightly to the left due to the enough adsorbing concentration of PGPR. It was in line with the findings of Marze (26) that slightly higher PGPR concentrations were sufficient for interface coverage 1.2 mg/m^2^. It has even been reported that larger inner water droplets could lead to the full release of the internal droplet content into the external water phase (27). It was consistent with those previously reported by Hattrem et al. (28) and Serdaroglu et al. (29) for slightly higher PGPR concentrations with good stability of double emulsion.

#### Zeta Potential

The effect of different concentrations of PGPR on the zeta potential of *Monascus* pigment double emulsion is shown in Figure 2C. It was found that the zeta potential of all *Monascus* pigment double emulsions was negative, indicating the negatively charged emulsions droplets. The zeta potential was obviously affected by the content of PGPR used for emulsification. As shown in Figure 2C, the magnitude of the zeta potential of the *Monascus* pigment emulsion was decreased gradually from −32.0 to −20.0 mV with increasing PGPR concentration from 0.9 to 5.4 wt%. This might be explained by the fact that PGPR is a non-ionic surfactant; therefore, a decreased effect on zeta potential was observed by the droplets surrounded by non-ionic surfactants (30). The results suggested that emulsions showed gradual tendency of adsorption with increasing PGPR content. It was found that there was no significant difference between zeta potential of emulsions with more than 3.6 wt% PGPR. It indicated that enough concentration of PGPR for preparing *Monascus* pigment double emulsion was 3.6 wt%.

#### Physical Stability

To further investigate the influence of PGPR concentration on the physical stability of *Monascus* pigment double emulsions, the instability index was present (Figure 2D). The higher instability index exhibits the much lower stability of the emulsion. As can be seen, the instability index of *Monascus* pigment double emulsions decreased with increasing PGPR concentration. It demonstrated that preparing *Monascus* pigment double emulsions with high concentrations of PGPR could enhance the stability. The improved stability of *Monascus* pigment double emulsions with increasing content of PGPR can be attributed to the strong steric repulsion between droplets resulting from the relatively thick PGPR layer adsorbed to their surfaces. *Monascus* pigment double emulsions with high concentrations of PGPR had high initial osmotic pressure; a good physical stability could be achieved providing that the PGPR concentration is sufficient (31). On the other hand, the improved stability of the *Monascus* pigment double emulsions can also be attributed to the increased viscosity and a gel-like network. Similar results were also reported by Cofrades et al. (32) and Serdaroglu et al. (29) who proved that 6.0–6.4 wt% PGPR in the oil phase was stable on the double emulsions used as animal fat replacers in meat systems.

#### Microstructure

**Figure 3:**
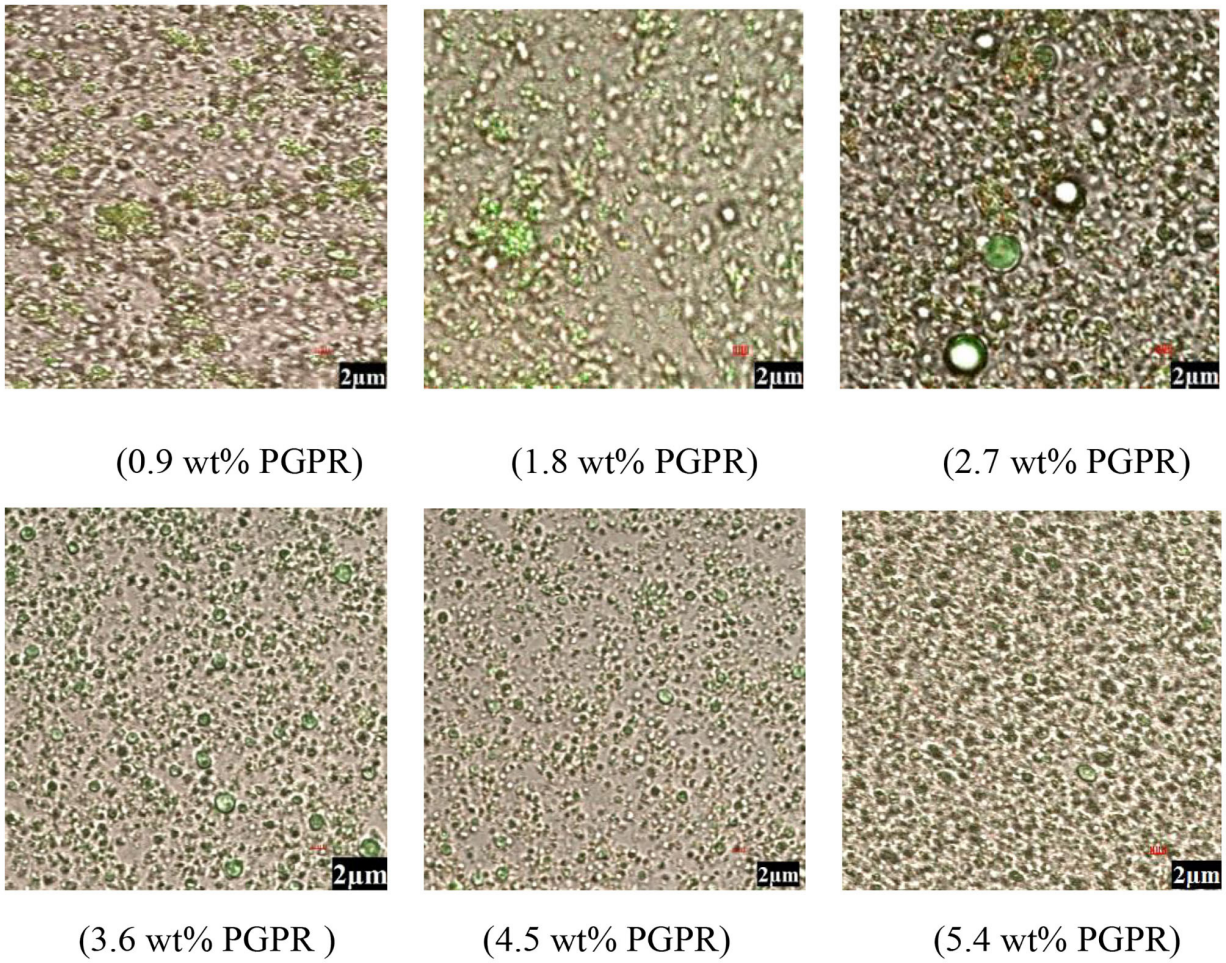
Microscopic images of *Monascus* pigment double emulsions with different PGPR concentrations.

Confocal laser microscopy images of *Monascus* pigment double emulsions with different PGPR concentrations are shown in Figure 3. In the images, the green area represented oil phase enrichment and the red area represented the *Monascus* pigment phase region. In the microstructure observed by CLSM, *Monascus* pigment in the out water phase could be seen in the double emulsion with 0.9 wt% PGPR and some aggregated droplets were shown in the emulsions prepared with 0.9, 1.8, and 2.7 wt% PGPR. As the PGPR concentration was increased (3.6 and 4.5 wt%), aggregated droplets in the microstructure disappeared and small droplets were evenly distributed throughout the emulsion system. It was found that some droplet aggregation was also observed at relatively higher PGPR (5.4 wt%) concentrations. It can be concluded that the microstructure of *Monascus* pigment double emulsion stabilized with 3.6 and 4.5 wt% PGPR was more uniform. According to Eisinaite et al. (31), the water phase was entrapped in the PGPR formed reverse micelles. Therefore, when the content of PGPR was lower, the interfacial tension reduction and the increase of the polarity of the oil phase were not enough to prevent the aggregation of droplets (33). This phenomenon suggests that the presence of enough PGPR could improve the stability of *Monascus* pigment double emulsion against aggregation and coalescence.

Therefore, 3.6 wt% PGPR was the optimum concentration for preparing stable *Monascus* pigment double emulsions. Because the droplets had a relatively smaller droplet size, high zeta potential, and a lower instability index, the *Monascus* pigment double-emulsion droplets appeared to be saturated with PGPR at this concentration.

#### Microrheological Properties

**Figure 4:**
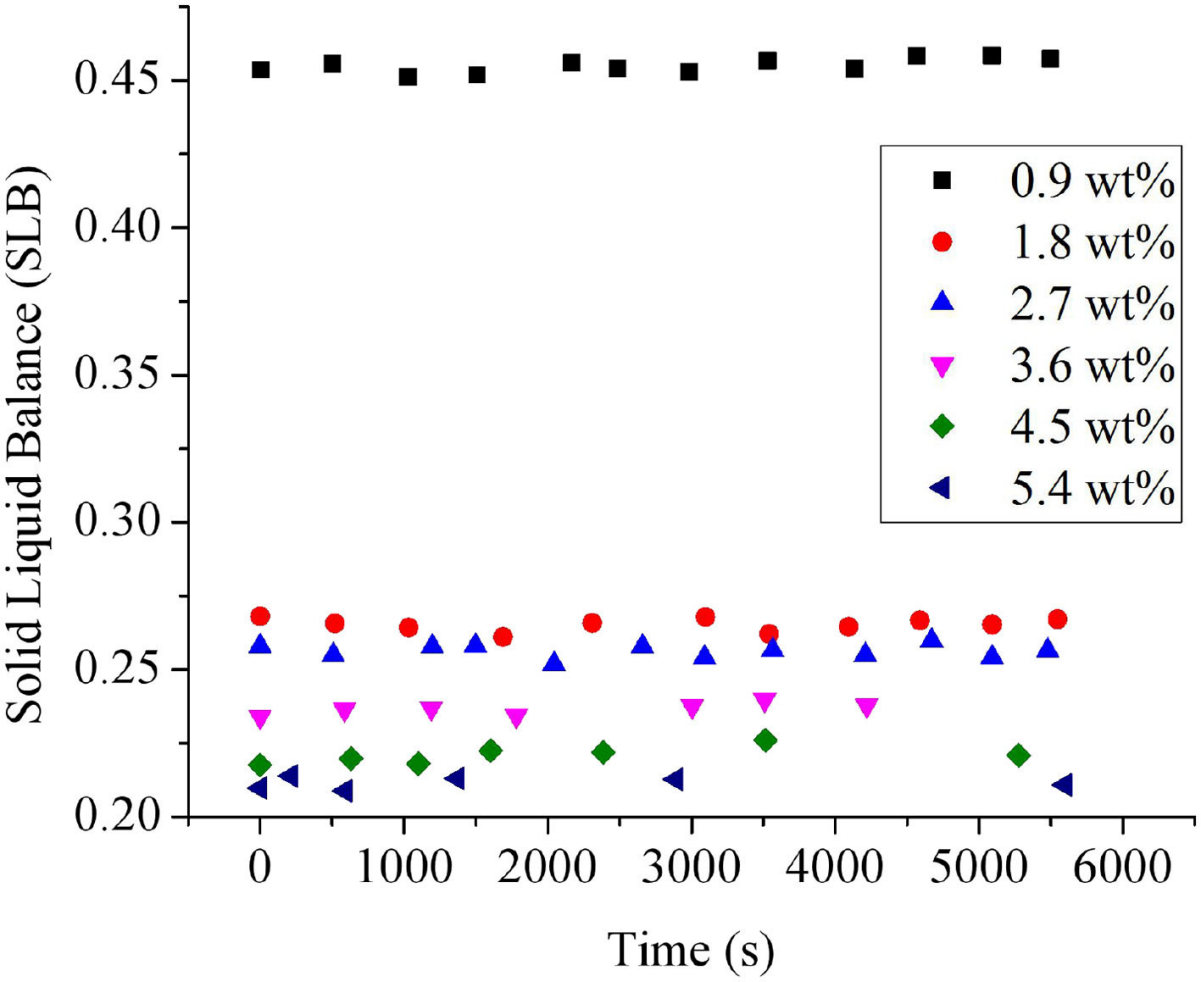
Influence of PGPR concentrations on the solid–liquid balance (SLB) values of *Monascus* pigment double emulsions.

The influence of PGPR concentrations on the microrheological properties of *Monascus* pigment double emulsions was measured using the Rheolaser lab. The technique monitored the Brownian motion and the interactions of droplets (34). The microrheology study could also illustrate the solid and liquid characteristics of emulsion by solid–liquid Balance (SLB) value, which is a ratio between the solid-like and liquid-like behavior of the sample (23). The droplets' movement showed a more solid behavior when the SLB is <0.5, while the emulsion showed a more viscous or liquid behavior with the SLB ranging between 0.5 and 1 (35). As shown in Figure 4, all the SLB values of *Monascus* pigment double emulsions were <0.5, which means that the solid behavior dominated at all the PGPR concentrations. The SLB values of *Monascus* pigment double emulsions were decreased with increasing PGPR content. It suggested that the elastic characteristic of the *Monascus* pigment double emulsion was enhanced by the further addition of PGPR. It was mainly due to the increased collision and interaction between the droplets during the movement resulting in force increasing. These results could be explained in terms of a decrease in the mobility of the *Monascus* pigment double-emulsion droplets with increasing PGPR concentration. It proved that enough PGPR did adsorb to *Monascus* pigment double-emulsion droplets, leading to a dramatic decrease of droplet mobility compared with the lower content of PGPR.

### Effect of SPI on the *Monascus* Pigment Double Emulsions

#### Particle Size and Zeta Potential

**Figure 5:**
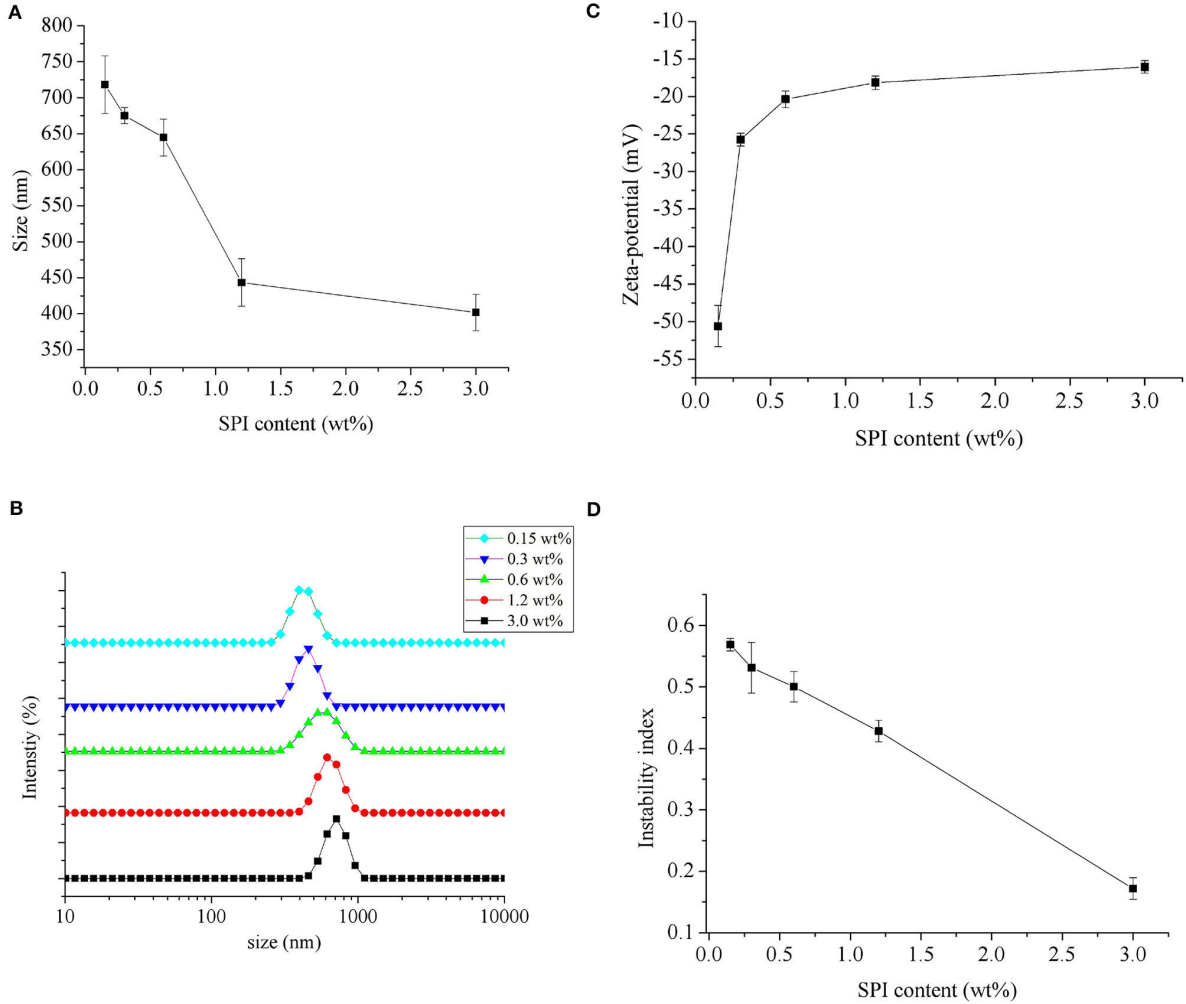
The mean particle size **(A)**, particle size distribution **(B)**, zeta potential **(C)**, and instability index **(D)** of *Monascus* pigment double emulsions with different SPI concentrations.

The impact of varied SPI contents on the average droplet size and droplet size distributions of *Monascus* pigment double emulsion is investigated in Figure 5. It was found that SPI concentration had a strong influence on the droplet size of *Monascus* pigment emulsion. With increasing SPI concentration, smaller droplets were formed, which is in accordance with conventional emulsification theory (36). The stabilizing effect of emulsifier molecules increases with its concentration. When the SPI concentration was 3.0 wt%, there was a large decrease in the mean particle size. The decrease in droplet size was a clear indication of the enough adsorption of SPI onto the *Monascus* pigment double-emulsion droplets and corresponded to the differences in the droplet charge described in Figure 5C. Therefore, SPI as a surfactant was adsorbed to the surface of the droplets forming a protective coating that inhibits droplet aggregation.

Zeta potential, besides size distribution, is another important parameter characterizing stability of emulsion. Figure 5C shows the influence of SPI concentration on the zeta potential of *Monascus* pigment double emulsions. It was found that the net charge on the *Monascus* pigment double emulsions became less negative as the SPI was increased. It is generally known that the surface charge of the protein-coated emulsion droplets is governed by the degree of ionization of amino groups (–NH_2_) and carboxyl groups (-COOH) of the protein molecules. The decrease of zeta potential of *Monascus* pigment double-emulsion droplets with increasing SPI content was attributed to that the structure of interfacial SPI was altered due to its adsorption (37).

#### Physical Stability

*Monascus* pigment double emulsions stabilized with 0.15–3.0 wt% SPI were examined by the instability index according to the integrated transmission profiles against the measuring time in Figure 5D. As the SPI concentration increased, the instability index decreased, suggesting that SPI can protect the *Monascus* pigment emulsion from flocculation. The addition of SPI had an optimum effect on the stability for *Monascus* pigment emulsions with 3.0 wt% SPI, since the highest SPI concentration led to the lowest instability index. It was reported that the addition of biopolymers to the external phase improved physical stability by forming a coating around droplets or increasing the viscosity (38). The results could be explained by the fact that when the SPI concentration was low, it was insufficient to cover the entire droplet in the emulsion. Thus, it may lead to bridge flocculation by interacting the surfaces of the *Monascus* pigment double emulsions through attractive electrostatic interaction. With increasing concentration of SPI, it would be enough to cover the droplets and form a thick layer on the *Monascus* pigment double-emulsion droplets. Therefore, it would inhibit the flocculation by the steric hindrance repulsion.

#### Microstructure

**Figure 6:**
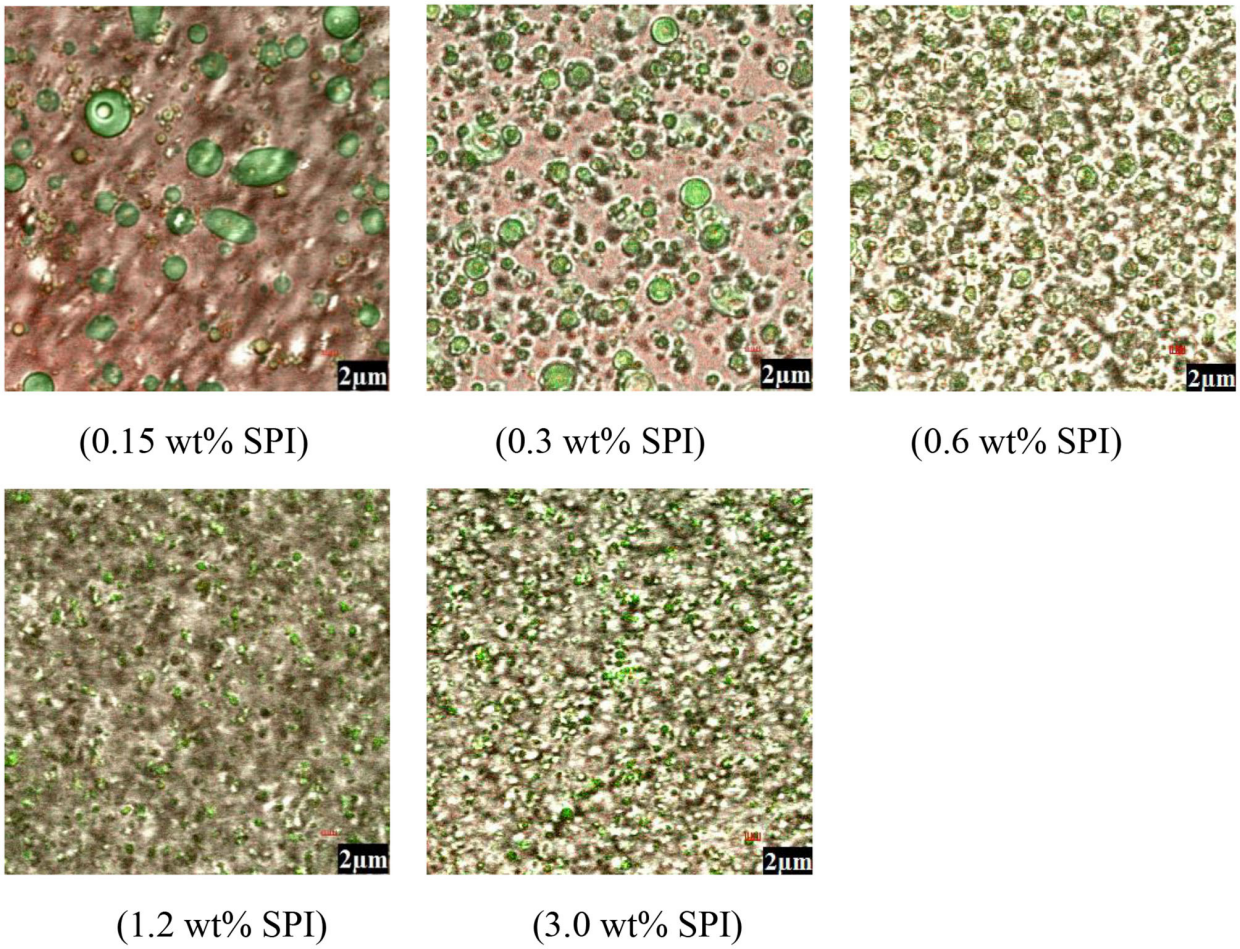
Microscopic images of *Monascus* pigment double emulsions with different SPI concentrations.

The appearance of *Monascus* pigment double-emulsion droplets was observed by confocal microscopy as shown in Figure 6. The CLSM images confirmed the formation of *Monascus* pigment double emulsions, which consist of W/O emulsion droplets in the continuous aqueous phase. The oil droplets dyed with Nile red appeared green, the SPI molecules dyed with Nile blue appeared red, and the inner water phase without dye appeared white. When the W_2_ phase was at low (0.15 and 0.3 wt%) SPI concentration, the double emulsions consisting of relatively large oil droplets without uniform sizes, with some smaller water droplets inside, gave a visual impression of inhomogeneity. The particle size of *Monascus* pigment double emulsions was decreased with SPI concentration increasing from 0.6 to 3.0 wt% leading to the reduction in coalescence of W/O emulsion droplets. It may be because SPI exhibit O/W droplets a layer with steric hindrance interaction, as confirmed by the observation of double-emulsion droplets.

#### Microrheological Properties

**Figure 7:**
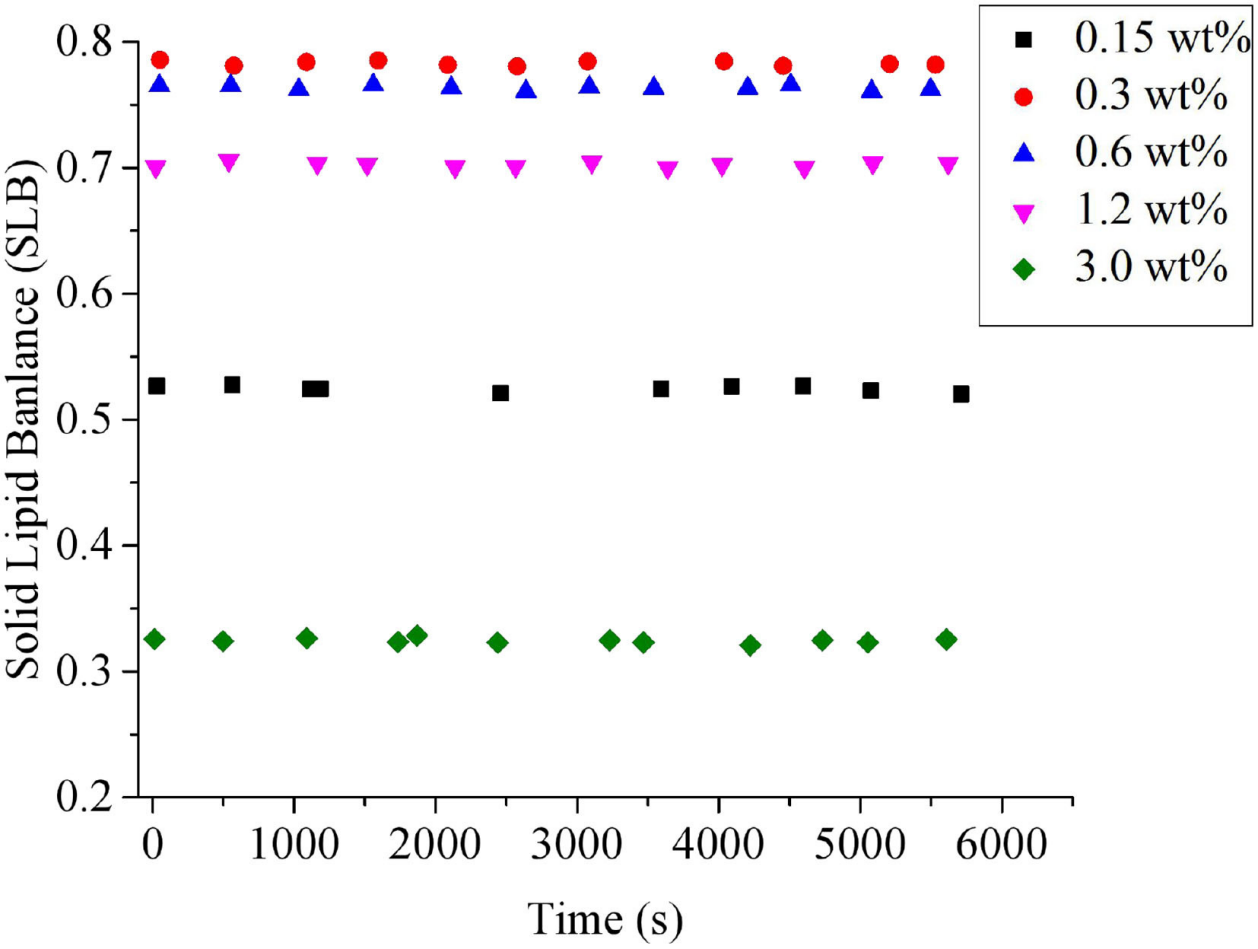
Influence of SPI concentrations on the solid–liquid balance (SLB) values of *Monascus* pigment double emulsions.

The effect of SPI concentration on the microrheological property of *Monascus* pigment double emulsion was investigated without disturbing the emulsions system by using the Rheolaser lab. The SLB value indicates the equilibrium state of the emulsion by calculating the slope of the platform area (39). In Figure 7, it was found that the SLB values of the double emulsions were more than 0.5 with SPI <3.0 wt%, which indicated that those emulsions were liquid behavior, while there was a sharp decrease in SLB value as the SPI concentration was increased to 3.0 wt%. The SLB of *Monascus* pigment double emulsion stabilized with 3.0 wt% SPI was <0.5. It proved that the *Monascus* pigment double emulsion with 3.0 wt% SPI and 3.6 wt% PGPR presented a solid behavior leading to loss of droplet mobility. This phenomenon may be attributed to the colloidalization of the emulsion system as the SPI concentration was increased to a specific degree.

#### Effect of Ionic Strength, Heating, and Freeze Thawing Treatments on the Stability of Monascus Pigment Double Emulsion

**Figure 8:**
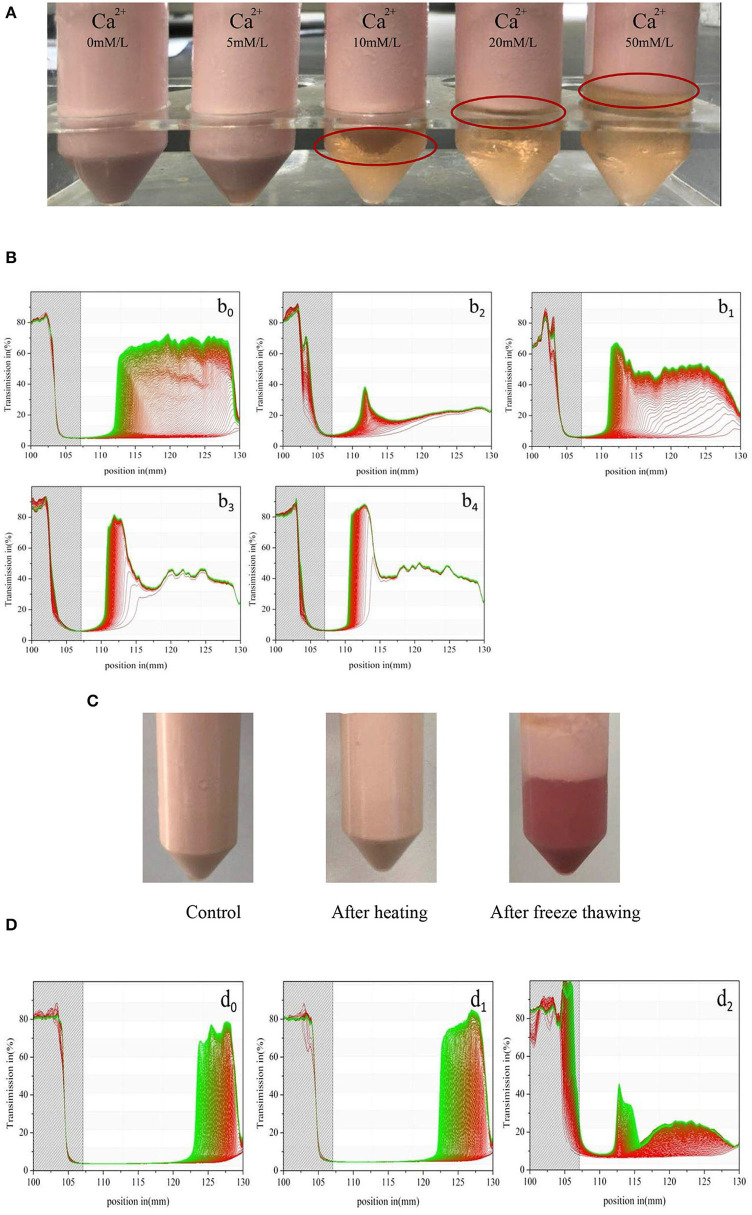
Effect of CaCl_2_ concentration on the visual creaming stability **(A)** and the original transmissions as a function of sample position of *Monascus* pigment double emulsions **(B)**. The stability was expressed as the slope of the integrated transmission time plots determined at 2,500 rpm during 7,650 s at 25°C (b_0_: without CaCl_2_; b_1_: with 5 mM CaCl_2_; b_2_: with 10 mM CaCl_2_; b_3_: with 20 mM CaCl_2_; b_4_: with 50 mM CaCl_2_). Effect of heating and freeze thawing treatment on the visual creaming stability **(C)** and original transmissions as a function of sample position **(D)** of *Monascus* pigment double emulsions (d_0_: control; d_1_: after thermal treatment; d_2_: after freeze thawing treatment).

Figure 8A shows the impact of different CaCl_2_ concentrations on the visual creaming stability and original transmissions as a function of the sample position of *Monascus* pigment double emulsions. According to the visual creaming stability, the increase of Ca^2+^ content in the systems produced a higher extent of creaming. It exhibited that *Monascus* pigment double emulsions with <5 mM CaCl_2_ prevented calcium to destroy the physical stability of emulsions, while *Monascus* pigment double emulsions with more than 10 mM calcium concentration were unstable.

The changes in the transmission profiles over space and time further prove that the influence of different Ca^2+^ concentrations on *Monascus* pigment double emulsions resulted in different physical stabilities. In Figure 8B, it was found that with increasing Ca^2+^ concentrations from 5 to 50 mM, the first profile taken after 30 s exhibited high transmissions along the sample length. A sharp front of *Monascus* pigment double emulsions with more than 10 mM moved toward the top during centrifugation. Our research group (40) reported that the sharp front means that nearly all droplets are moving as a zone creaming. The structure of the creaming was a flocculated network. With increasing concentration of the Ca^2+^ concentration more than 10 mM, the profiles were nearly spaced with a considerably smaller distance during centrifugation. It indicated that these *Monascus* pigment double emulsions with more than 10 mM Ca^2+^ were creaming easily. The profiles of *Monascus* pigment double emulsions with 20 and 50 mM Ca^2+^ were quite similar. This result can be explained by the binding of SPI by Ca^2+^, leading to the aggregation by electrostatic screening (41). It was reported that Ca^2+^ not only contributed its nutritional property but also acted functionally by allowing the obtaining of double-emulsion systems with creamy texture without the need of addition of saturated fats. The obtained result proved that the isolated creaming *Monascus* pigment double emulsions could work as a reduced fat replacer of whipped dairy cream with important calcium contribution.

The impact of heating and freeze thawing treatment on the *Monascus* pigment double emulsion was also studied. As shown in Figures 8C,D, it was found that there was no creaming and the *Monascus* pigment double emulsion was stable against heating treatment. During heating, the bonds between SPI molecules were formed. Presumably, hydrophobic interactions and disulfide bridges often play major roles (42). Hydrogen bonds may also be present. Roesch and Corredig (43) reported that covalent interactions have occurred between soybean proteins during heating. Therefore, heating could result in the improved viscosity of *Monascus* pigment double emulsion, which led to a more structured network.

After freeze thawing cycling, creaming occurred in *Monascus* pigment double emulsion. It indicated that the droplets migrated to the top of the sample and promoted their coalescence. It revealed that droplet aggregation and coalescence of *Monascus* pigment double emulsion occurred after freeze thawing treatments. The destabilization was attributed to the SPI relatively thin absorbed layers of the *Monascus* pigment double-emulsion droplets. It also might be because the adsorbed SPI either underwent a conformational change or is desorbed from the droplet surfaces due to freeze-thawing treatments (44).

## Conclusion

*Monascus* pigment double emulsions were fabricated in the presence of PGPR and SPI and characterized in terms of droplet size, stability, microstructure, and microrheological properties. According to the results, the droplet size as well as stability was highly dependent on the concentration of the PGPR and SPI used. Higher PGPR and SPI concentrations yielded lower droplet sizes and higher stability and more solid behaviors. It was shown that 3.6 wt% PGPR and 3.0 wt% SPI were the optimum concentrations of emulsifiers to form *Monascus* pigment double emulsion. Microstructure proved that aggregated droplets disappeared and small droplets were evenly distributed throughout the emulsion system with increasing PGPR and SPI contents. The elastic characteristic of the *Monascus* pigment double emulsion was enhanced by the addition of PGPR and SPI due to the increased collision and interaction between the droplets. *Monascus* pigment double emulsion was unstable to more than 10 mM Ca^2+^ and freeze-thawing treatment due to the weak electrostatic interaction. However, it was stable against heating treatment probably due to heating leading to a more structured network. It could be useful for a broad application of *Monascus* pigment double emulsions in food products.

## Data Availability Statement

All datasets generated for this study are included in the article/supplementary material.

## Author Contributions

DX, BZ, and YC: data curation, writing–original draft preparation, investigation, and validation. GL, YY, and SW: methodology, investigation, and validation. DX: writing–review and editing, project administration, and funding acquisition. YC: conceptualization, supervision, project administration, and funding acquisition. All authors contributed to the article and approved the submitted version.

## Conflict of Interest

The authors declare that the research was conducted in the absence of any commercial or financial relationships that could be construed as a potential conflict of interest.
